# Editorial: Dendritic Spines: From Biophysics to Neuropathology

**DOI:** 10.3389/fnsyn.2021.652117

**Published:** 2021-02-18

**Authors:** Martine Ammassari-Teule, Carlo Sala, Menahem Segal

**Affiliations:** ^1^Istituto di Biochimica e Biologia Cellulare, National Research Council (CNR) and Fondazione Santa Lucia, Rome, Italy; ^2^Neurobiology Department, National Research Council (CNR) Neuroscience Institute, Milan, Italy; ^3^Neurobiology Department, Weizmann Institute, Rehovot, Israel

**Keywords:** dendritic spine, calcium, Alzheimer's disease, plasticity, hippocampus

The molecular and cellular mechanisms that govern dendritic spine formation, modification, and elimination have been the focus of tremendous interest in recent years. Using an advanced arsenal of molecular and high-resolution imaging methodologies, the analysis of dendritic spine functions becomes more complex and detailed. The striking morphological heterogeneity of adjacent spines on the same dendritic branch and their minute size prohibits the structure/function analysis of spines. Despite the growing number of publications (more than 10,000 to date), major issues concerning the molecular, cellular, and functional attributes of spines remain unsettled. Are there different molecular pathways for different spines? Are there structural correlates of different classes of memories (e.g., episodic, semantic, short, and long term)? What happens to the structural change when a memory is forgotten? Can we improve memory by changing the structure? These and many other issues are still open. Every so often one needs to pause and review the literature to see how far we have advanced and how far away we are from understanding spines in relation to growth, maintenance, and deterioration of brain functions.

This Research Topic on *Dendritic Spines From biophysics to Neuropathology* addresses these issues. It includes four extensive review articles and five original research articles written by leading groups in the field cover subjects ranging from biophysics and molecular biology to functional and network attributes of spine functions. The reviews address standing issues in the study of spines. For example, Pchitskaya and Bezprozvanny propose to replace the standard classification of spine categories into mushroom, stubby, long, and filopods to a continuum of spine shapes. This new proposal is logical in view of the current inability to associate distinctly separate functions to different shapes of spines, but it requires more analytical tools to characterize the exact shape of a given spine.

Another review, by Runge et al., attempts to link spine dynamics with circuit rewiring in relation to external signals that generate these network changes found in neuropsychiatric disorders. They list two-photon microscopy (TPM) as a leading tool in the attempt to track changes in spines in the living brain. Studies using TPM have made a great contribution to the analysis of spine function, including the assertion that spines are transient and do not last throughout the life of the organism, but that their turnover time is different for different structures. This brings back the question of whether the individual spine is the locus of memory.

Ammassari-Teule takes us to the pathological end of spine functions. Therapeutic attempts to cure Alzheimer's disease (AD) have failed in the past decade, suggesting that when behavioral symptoms appear, the brain is already affected by the disease in an irreversible manner. Thus, the idea is to detect early signs of neuropathology and continue from there. It has been suggested in mouse models of AD that the early changes are physiological—a reduction in ability to express long term potentiation (LTP). Ammassari-Teule then reviews studies that examine whether such changes can be detected in dendritic spines at the early stage of AD. The review focuses on changes in postsynaptic density as an early sign of AD, suggesting that synaptic dysfunction is a cause rather than a consequence of the cognitive decline associated with AD.

Finally, Costa et al. review the role of Rac GTPase in spine plasticity. They link the molecular, morphological, and functional aspects of GTPs in the brain. Rac GTPase is one of many chemicals associated with spine functions. They are able to show that altered Rac activity is associated with abnormal spine morphology and brain functions, including neurodegeneration. This is an elegant link between a single small GTPase and an array of brain functions from the single memory to complex networks of the human brain.

The original research reports span the whole spectrum of methods and levels of analysis. Regulation of intracellular calcium ion concentration ([Ca^2+^]i), which has been known to regulate different forms of synaptic plasticity, is addressed by Jodar et al., studying olfactory bulb granular cell spines, and Kushnireva et al., studying calcium stores in cultured hippocampal neurons. SK channels, activated by calcium influx, regulate the excitability of dendritic spines and are shown to control spine plasticity via modulation of CaMKII (Shrestha et al.). These three studies illustrate the wide spectrum of calcium regulation of dendritic spine plasticity. One interesting diversion from the traditional focus on intra-spine constituents is illustrated by the work of Nguyen et al., who analyzed the effects of variation in ephrinB1, a resident molecule in astrocytes, on dendritic spine structure and function in relation to learning and memory. Finally, de Schultz et al., expanded on their ongoing work on parent/offspring association in relation to long term effects of parental deprivation on dendritic spines in the medial prefrontal cortex. This work illuminates the role of parents and the long-lasting male/female difference in the effects of stress on the juvenile brain.

All in all, these studies highlight the current status of the major effort across the world on trying to understand the roles of dendritic spines, the minute but extremely important neuronal organelle, in the regulation of brain function. Over the years, this tiny structure turned out to be an extremely complex organelle, where over 500 different types of molecules coordinate to regulate its structure and function. Many issues remain, including the tremendous heterogeneity of adjacent spines on the same dendrite, the fast turnover rate of spines in different behavioral states, and the cardinal issue of whether a given spine is the locus of memory. These and many more issues should be addressed in future studies using more advanced molecular tools.

## Author Contributions

All authors listed have made a substantial, direct and intellectual contribution to the writing of this Editorial, and approved it for publication.

## Conflict of Interest

The authors declare that the research was conducted in the absence of any commercial or financial relationships that could be construed as a potential conflict of interest.

